# Novel compound heterozygous *FAM20C* variants cause Raine syndrome – retrospective prenatal diagnosis and literature review

**DOI:** 10.1186/s13023-026-04333-2

**Published:** 2026-03-27

**Authors:** Ewelina Lazarczyk, Maria Pilarska-Deltow, Anna Sowinska-Seidler, Anna Repczynska, Malgorzata Drozniewska, Aleksander Jamsheer, Agata Zdrojewska, Magdalena Pasinska, Olga Haus

**Affiliations:** 1 Department of Clinical Genetics, Faculty of Medicine, Collegium Medicum, Bydgoszcz, Nicolaus Copernicus University, Torun, Poland; 2 Department of Medical Genetics, Collegium Biologicum, Medical University, Poznan, Poland; 3 Synnovis Analytics LPP, Guy’s Hospital, London, UK; 4 Neonatology Clinic of the University Hospital, No. 2, Bydgoszcz, Poland

**Keywords:** Array comparative genomic hybridization (aCGH), Prenatal diagnosis, Raine syndrome

## Abstract

**Background:**

Raine syndrome, RS, (OMIM 259775) is a rare autosomal recessive disorder with prevalence of less than 1:1 000 000, caused by homozygous or compound heterozygous variants in *FAM20C* gene. A retrospective genetic investigation was performed on DNA extracted from amniotic cell cultures previously used for cytogenetic studies.

**Methods:**

Extracted DNA was used for aCGH (array comparative genomic hybridization) and Sanger sequencing. Parental blood samples were tested for karyotype (GTG – G-banding using trypsin and Giemsa) and molecular karyotype (aCGH). Additionally, paternal sample was tested by NGS (next generation sequencing).

**Results:**

We present two fetal cases of Raine syndrome. Both were compound heterozygotes for two *FAM20C* gene variants: a maternally-inherited copy-number loss encompassing exons 1-3 (arr[GRCh37] 7p22.3(170366_229852)x1) and a paternally-inherited novel frameshift exon 1 variant [NM_020223.4:c.307_308dupTC p.(Ser104ArgfsTer27)].

**Conclusion:**

Prenatal phenotype associated with Raine syndrome often includes characteristic pattern of intracranial calcification, osteosclerosis and facial dysmorphism. However, in majority of cases, diagnosis is made postnatally. It is therefore important to report all cases of Raine syndrome for which USS (ultrasound scan) findings are available – this will enable better understanding and detection of RS prenatally.

**Supplementary Information:**

The online version contains supplementary material available at 10.1186/s13023-026-04333-2.

## Background

Raine syndrome, RS, (OMIM 259775, ORPHA1832) is a rare autosomal recessive disorder with prevalence of < 1:1 000 000 [1, 2]. It was first reported in 1985 by Whyte et al. as congenital sclerosing osteomalacia with cerebral calcification [3]. In 1989 Raine et al. reported a newborn with osteosclerosis, facial dysmorphism and lethal skull anomalies. In 1992 the disorder was submitted to OMIM as Raine syndrome (lethal osteosclerotic bone dysplasia) [2, 3]. Identification of homozygous and compound heterozygous variants in *FAM20C* gene, alongside with detection of pseudodicentric chromosome 7 [45,XY, psu dic(7;7)(p22;p22)] coexisting with biallelic loss of *FAM20C* in one patient led to description of genetic background of this syndrome by Simpson et al. in 2007 [4].

*FAM20C* gene (located at chromosome 7p22.3) includes 10 exons and encodes FAM20C protein (a Golgi localised serine kinase). This protein is a member of FAM20 protein family that is required for osteoblast differentiation and mineralisation. The encoded protein binds calcium and phosphorylates proteins involved in bone mineralization, including fibroblast growth factor 23 and regulators of teeth and bone hard tissue formation and bone, dentin and enamel mineralization [2].

Various types of pathogenic variants harbouring different exons of *FAM20C* have been detected so far and, although no definitive gene variant – phenotype correlation has been established [2, 5], generalised osteosclerosis, facial dysmorphism and intracranial calcifications are indicated as the most frequent features of RS [2, 5, 6]. In 2009 Simpson et al. reported two unrelated patients with mild RS phenotype – this led to recognition of two RS subtypes (according to survival): lethal and non-lethal [2, 7].

### Clinical report

**Fig. 1:**
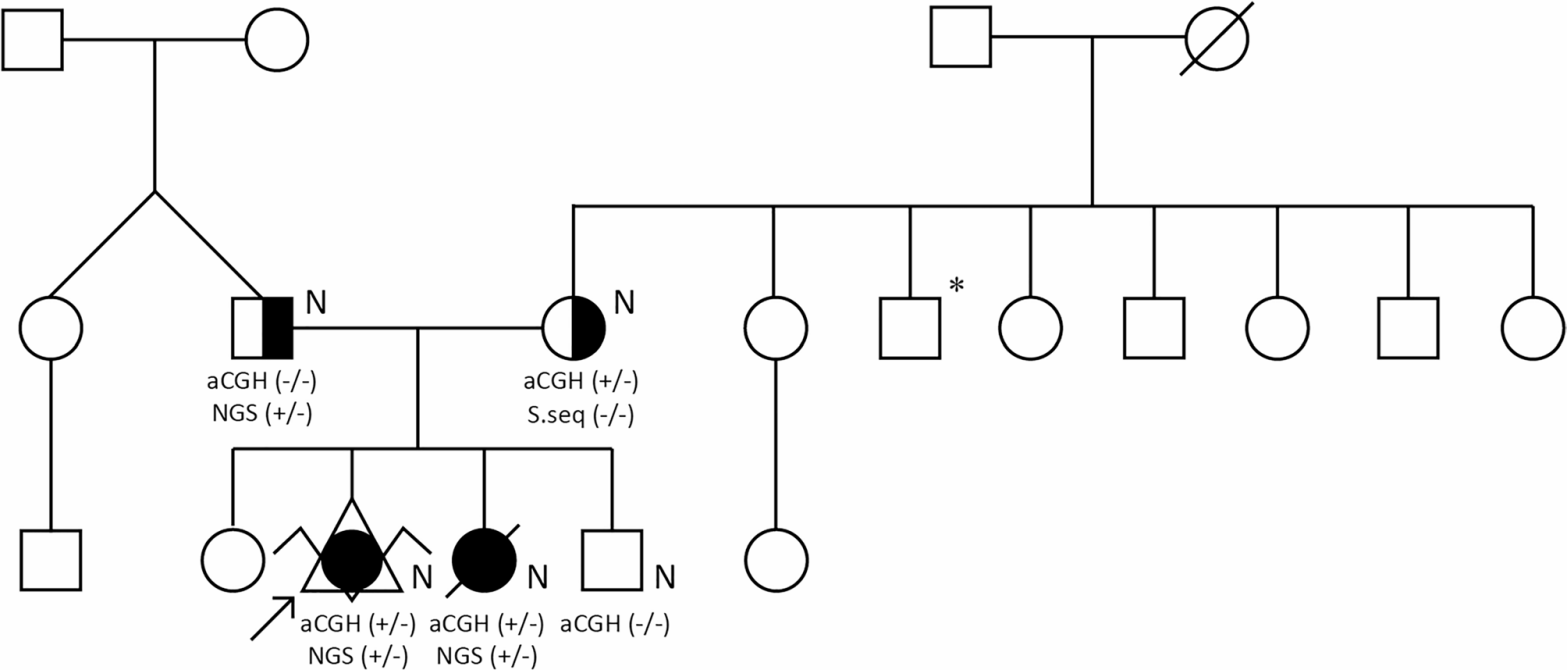
Pedigree of the examined family. Square – male; circle – female; symbol with diagonal line – deceased; black symbol – affected individual; half-filled symbol – carrier; arrow – proband; N – normal karyotype; asterisk (*) – memory problems; aCGH (-/-) – no 7p22.3 loss; NGS (+/-) – heterozygous c.307_308dupTC variant; aCGH (+/-) – heterozygous 7p22.3 loss; S.seq (-) – no *FAM20C* c.307_308dupTC in Sanger sequencing

The couple have not reported consanguinity and had two healthy children born from first and fourth pregnancy. Multiple anomalies were detected in second and third pregnancy. Pedigree of the examined family is shown in Fig. 1.

### Clinical report (Pregnancy II)

**Fig. 2:**
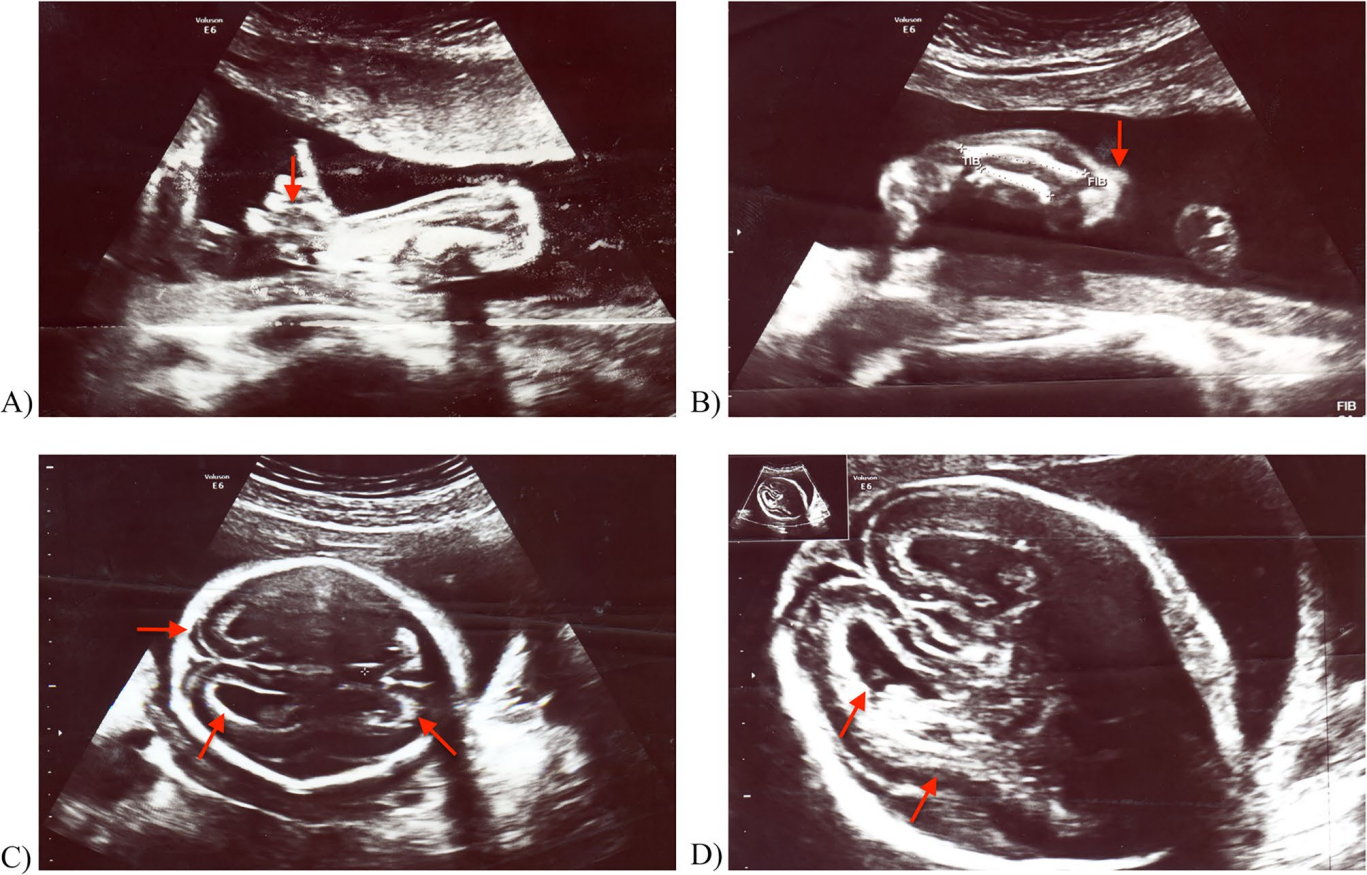
USS images taken at 20 weeks gestation. (**A**) Right hand - abnormally positioned thumb. (**B**) Thickening of periosteum, metaphyseal sclerosis, bowing and shortening of the long bones of the lower limb. (**C**) Increased density of the skull bones and the periventricular area of ​​the anterior and posterior horns of the lateral ventricles. (**D**) Increased density of skull bones and white matter in the periventricular area of ​​the posterior horns of the lateral ventricles, especially on the right side

Ultrasound scan (USS) performed at 20 weeks gestation (fetal weight – 312 g) revealed number of anomalies including hyperechogenicity of the skull with a very narrow septum pellucidum cavity. The width of the posterior horn of the left lateral ventricle was 8.6 mm. The walls of the lateral and posterior ventricles showed presence of calcifications in the periventricular white matter and in the choroid plexuses with significantly increased echogenicity (Fig. 2C and D). Hypertelorism with proptosis were present. The fetus had abnormal facial shape, single-chamber nostrils, no visible nasal bone, and turribrachycephaly. Narrow chest pressing on the lungs was noted. The heart had four chambers of a symmetrical structure with its axis shifted to the left. Abdomen was protruding above the chest line. There was a delay of approximately 3 weeks in the ratio of chest circumference to abdominal circumference. Bones of the lower legs had thickened periosteum and metaphyseal sclerosis, and were bent in the form of a headphone and shorter by about 3 weeks in relation to the gestational age (Fig. 2B). There was an increased echogenicity of distal bone sections. The hands were clenched into fists, with thumbs sticking out (Fig. 2A). During the examination, the volume of amniotic fluid was normal. Figure 2 shows images taken during at the above-described USS.

The pregnancy was terminated at 21 week gestation.

### Clinical report (pregnancy III)

The patient fell pregnant 12 months after termination of the second pregnancy. USS at 13 + 6 weeks did not reveal any obvious anomalies. The results of first trimester screening indicated low risk for trisomy 21 (1:20 000) and trisomy 18 (1:100 000). Fetal weight at 20 weeks was 370 g, sex was difficult to ascertain. USS showed ventriculomegaly, multiple sites of periventricular brain calcification, hemorrhages in encephalon and soft skull bones with lemon sign. Additionally, hypertelorism and exophthalmos were noted, whereas nasal bone was not visualised and the nose appeared abnormal. The chest was narrow. The heart had four chambers, and the apex pointing to the left.

USS performed at 28 weeks showed abnormal shape of the skull bones, trigonocephaly, narrow frontal suture, narrow forhead with preserved coronal suture, widening of the anterior and posterior horns of the lateral ventricles, increased echogenicity of the periventricular area, abnormal fetal profile with a retracted midface, shallow eye sockets with two symmetrical eyeballs, bilateral nasal bones, cleft upper lip and palate, invisible anterior part of the nasal septum, protruding tongue. The chest was bell-shaped with significantly protruding stomach.

At 31st week of gestation the pregnancy ended with a premature birth of a baby girl weighing 1620 g. She was scored 1 point in Apgar scale and died 4 h after being born.

Polyhydramnios was noted shortly before delivery.

**Fig. 3:**
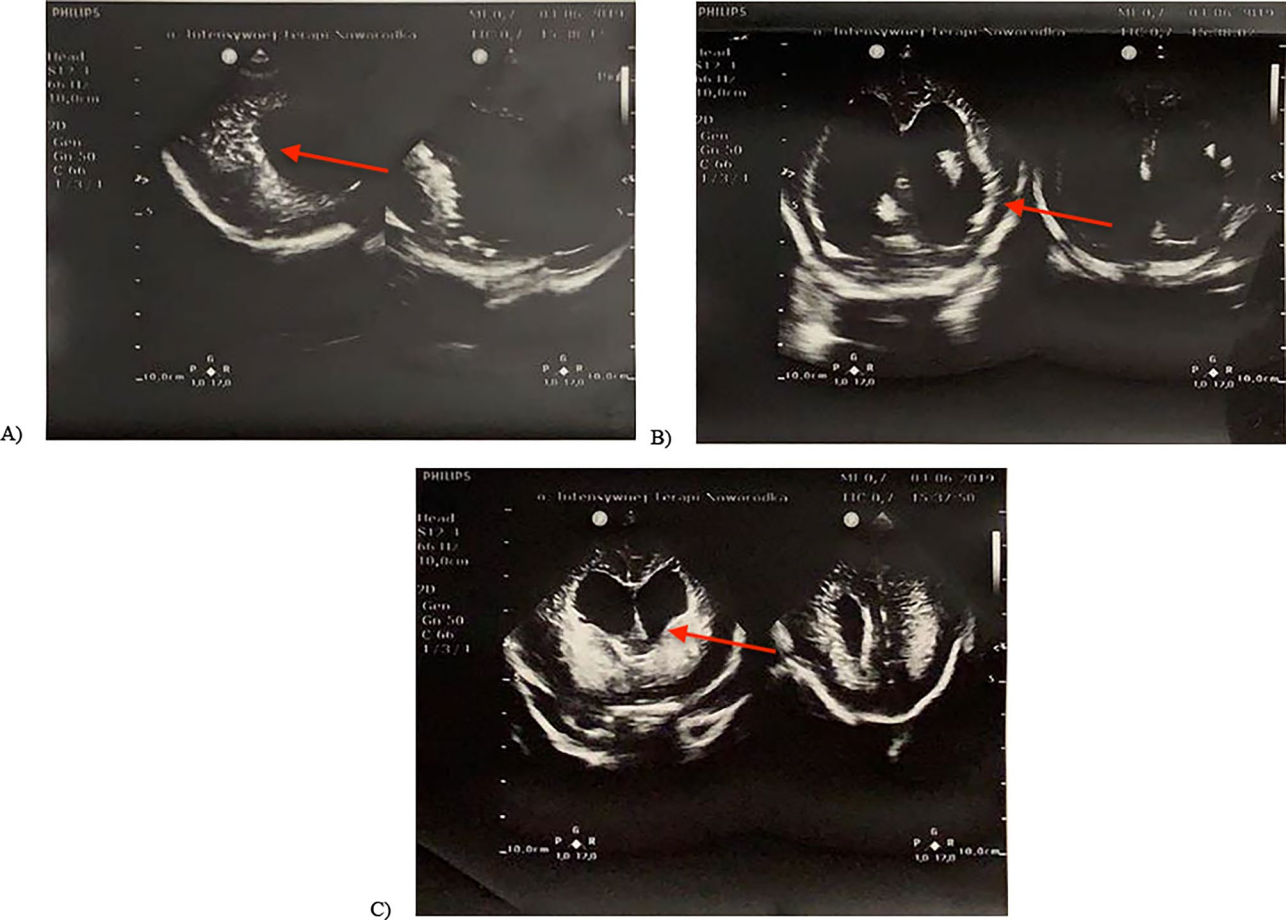
Images from transfontanellar USS in child III. (**A**) Increased bone density of the cranial vault and base of the skull. (**B**) Focal cerebral calcifications in the periventricular white matter and basal ganglia, as well as some calcifications of the meninges. (**C**) Hydrocephalus. Enlargement of the third and fourth ventricles of the brain

Postnatal assessment of clinical features revealed triangular-shape skull, large midline bone loss (in forhead – occipital axis), conjunctival hemorrhages, large and protruding tongue, underdeveloped and depressed nasal area, cleft of the upper and lower alveolar processes, esophageal obstruction, bell-shaped chest with large, bloated and hard stomach. Transfontanellar ultrasound revealed dilated cerebral ventricles, with increased ossification of skull base, as well as the facial bones and brain structures (Fig. 3).

## Materials and methods

Prospective karyotype analysis was performed on amniotic fluid samples after cell cultures. Retrospectively, cell suspension was used for DNA extraction for aCGH (array comparative genomic hybridization) and Sanger sequencing. Parental samples were used for karyotype analysis and DNA extraction for aCGH, NGS (next generation sequencing) and Sanger sequencing.

### Cytogenetics

Cytogenetic analysis was performed on cultured amniocytes (pregnancies II, III and IV) and lymphocytes obtained from peripheral blood (both parents). Karyotypes were described according to International System for Human Cytogenomic Nomenclature 2024 (ISCN) [8].

Amniotic fluid cells were cultured in parallel in AmnioGrow Plus Medium (CytoGen) and AmnioMAX II Complete Medium (Gibco) for 14–21 days. Postnatal karyotype was analysed after standard lymphocyte culture for 72 h.

### DNA extraction

DNA was extracted with QIAamp^®^ DNA Blood Mini Kit (Qiagen) according to manufacurer’s instructions. In case of DNA extraction from cell suspension, the suspension was washed with PBS. The procedure was then followed by instructions provided by manufacturer.

### Array comparative genomic hybridization

aCGH analysis was performed on SurePrint G3 Human CGH ISCA v2 8 × 60 K platform (Agilent Technologies, Santa Clara, California, USA) following manufacurer’s protocol. Scanning was performed with SureScan Dx Microarray Scanner (Agilent Technologies) and data was processed with Agilent Feature Extraction for CytoGenomics 4.0.1.21. Analysis was performed with Agilent CytoGenomics Software 5.0.0.38. aCGH was performed on DNA from both parents and from pregnancies II, III and IV. The following databases were used during the analysis: DGV (http://dgv.tcag.ca/dgv/app/home), DECIPHER (https://www.deciphergenomics.org/), UCSC (https://genome.ucsc.edu/), ClinGen (https://genome.ucsc.edu/), dbVar (https://www.ncbi.nlm.nih.gov/dbvar), ClinVar (https://www.ncbi.nlm.nih.gov/clinvar/), OMIM (https://omim.org/), PubMed (https://pubmed.ncbi.nlm.nih.gov/).

### Targeted next-generation sequencing

NGS was performed in paternal sample with a custom SureSelect gene panel (Agilent Design ID: 3060241, Agilent Technologies) targeting 77 genes associated with skeletal dysplasias. The panel was designed using SureDesign software (Agilent Technologies).

1 µg of high-molecular DNA was used for the NGS library preparation and proceeded accordingly by means of hybrid capture-based target enrichment approach (Agilent Technologies) following the protocol described in Bukowska-Olech et al. [9]. The library was subsequently sequenced on the Ion GeneStudio S5 System (Thermo Fisher Scientific) using protocol provided by the manufacturer.

Raw sequence data were initially analysed using the Torrent Suite 5.18.1 software embedded within the Ion GeneStudio S5 System (Thermo Fisher Scientific). Accordingly, the sequenced reads were assembled and aligned to GRCh37 human reference genome. The called variants were annotated and analysed using the Ion Reporter Software from the Thermo Fisher Cloud (Thermo Fisher Scientific) and visually explored by means of the Integrative Genomics Viewer (IGV). The identified variant’s pathogenicity was evaluated by means of Varsome premium on-line tool and classified in accordance with the guidelines established by the American College of Medical Genetics and Genomics (ACMG) [10].

### Sanger sequencing

Sanger sequencing was performed in pregnancy II and III and in paternal sample.

Pathogenic variant was confirmed by Sanger sequencing (ABI3130) with BigDye Terminator v.3.1 Cycle Sequencing Kit and Xterminator (ThermoFisher, Waltham, MA, USA) according to manufacturer’s procedure. Specific primers were designed by Primer-BLAST tool as follows: 5’CCATGAAGATGATGCTGGTG and 3’CTGGTGTCGCTGTTCACATT.

## Results

Karyotypes of all family members were normal.

**Fig. 4:**
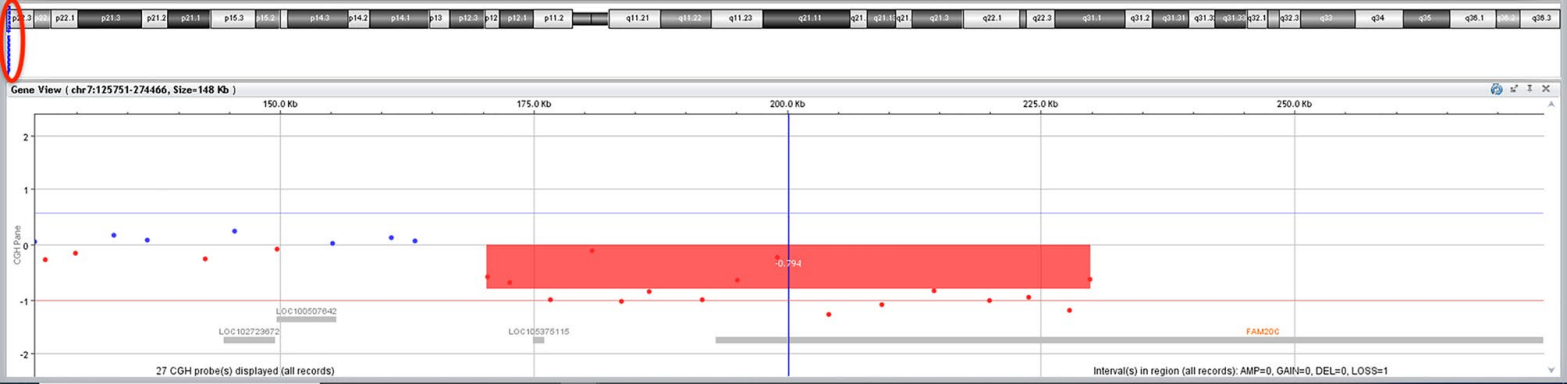
Partial aCGH result showing 7p22.3 deletion in fetus from pregnancy III. Red oval – indicates the position of the deletion on chromosome 7 ideogram

aCGH analysis revealed heterozygous deletion in chromosome 7 arr[GRCh37] 7p22.3(170366_229852)x1 of about 59 kbp in the mother and fetuses from pregnancies II and III (Fig. 4). The deletion included exons 1–3 of the *FAM20C* gene. The deletion was absent in the father and in the fetus from pregnancy IV. The variant was classified as likely pathogenic due to a significant gene-phenotype association, USS results being consistent with the clinical diagnosis, and the presence of a second variant in the *FAM20C* gene.

**Fig. 5:**
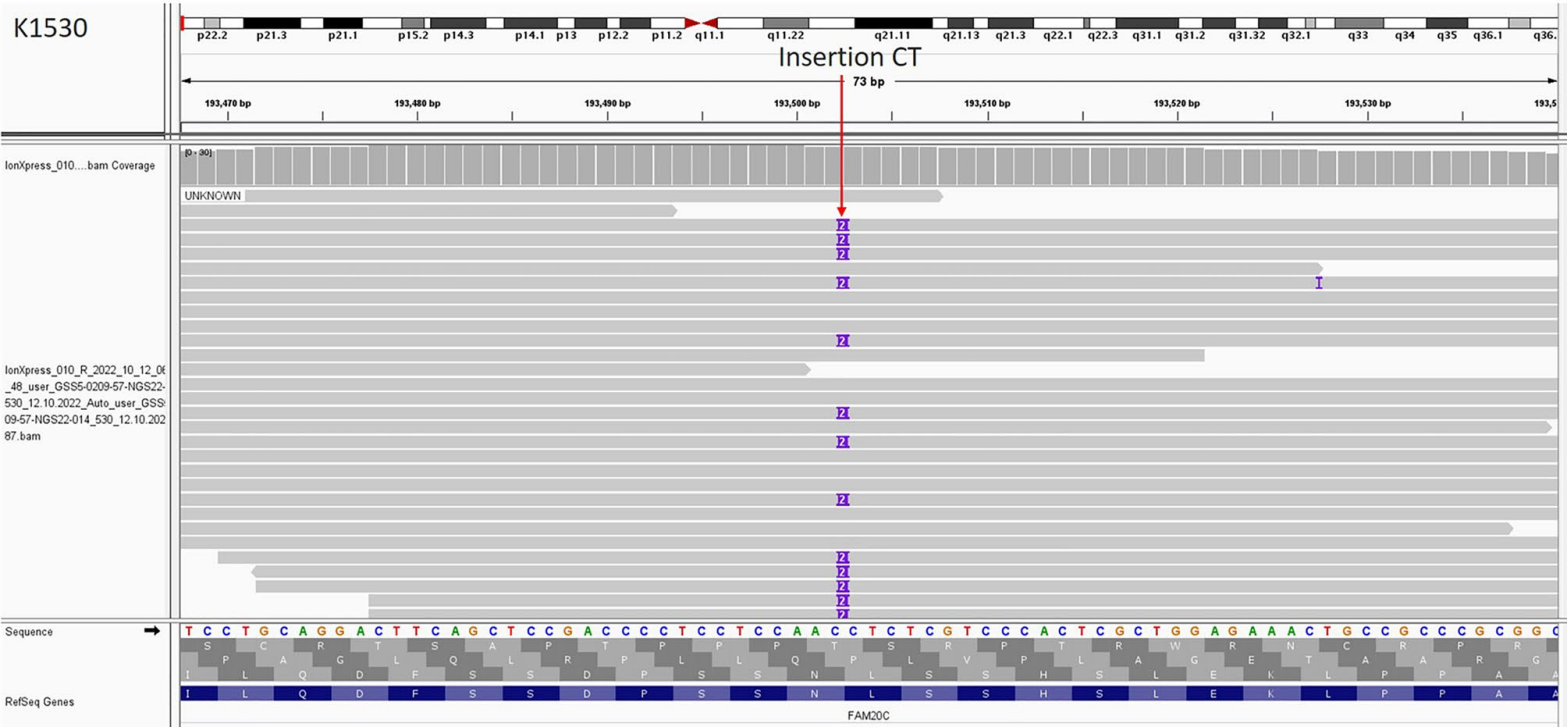
IGV visualisation of paternal heterozygous *FAM20C* variant (g.193506_193507dup)

**Fig. 6:**
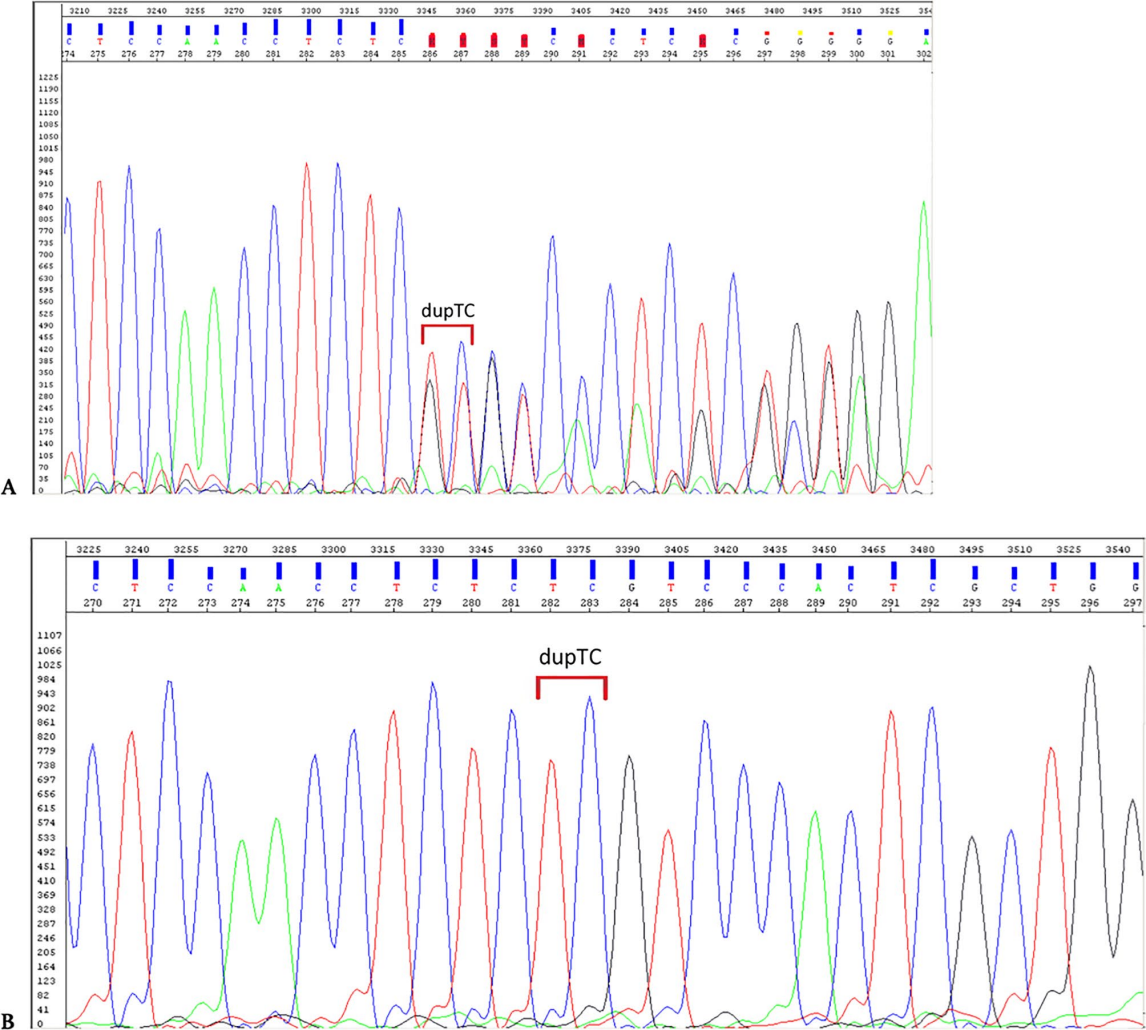
Electropherogram (**A**) of paternal NM_020223.4:c.307_308dupTC p.(Ser104ArgfsTer27), (**B**) of NM_020223.4:c.307_308dupTC p.(Ser104ArgfsTer27) in fetus from pregnancy II

Using the custom NGS gene panel performed on the father’s DNA we have identified a heterozygous duplication of two nucleotides in exon 1 of the *FAM20C* gene (NM_020223.4:c.307_308dupTC) (Fig. 5) leading to a truncation of the gene protein product p.(Ser104ArgfsTer27. The identified variant was absent in gnomAD Exomes v.2.1 and gnomAD Genomes v.2.1. The *FAM20C* variant was classified as pathogenic according the following ACMG criteria [10]: PVS1 Very Strong, PM2 Moderate, PP1 Supporting, PP4 Supporting, PM3 Moderate. The variant was covered by 28 uniquely mapping reads. We have not identified any other *FAM20C* variants that could be classified as pathogenic or likely pathogenic. The NGS results were confirmed by means of targeted Sanger sequencing (Fig. 6A).

It was also confirmed to be present in both affected fetuses (Fig. 6B) and absent in the mother. No NGS/Sanger sequencing were performed for children I and IV.

## Discussion

The application of molecular and cytogenomic testing in the reported family facilitated/enabled identification of genetic background of phenotypes observed in pregnancy II and III. Both fetuses were compound heterozygotes and their parents were carriers of two different *FAM20C* variants: 7p22.3(170366_229852)x1 (mother) and NM_020223.4:c.307_308dupTC p.(Ser104ArgfsTer27) (father).

As RS is inherited in autosomal recessive way, the risk for this couple of having similarly affected child in any consecutive pregnancy is 25%. Therefore, detailed scan, followed by prenatal genetic testing should be offered to the couple in any future pregnancies.

Both homozygous and compound heterozygous *FAM20C* single nucleotide variants (SNVs) have been identified in RS [2, 4, 6, 11]. Copy number variants **(**CNVs), however, are not a frequent finding. Similar (with regard to size and gene content) deletions have been recorded in ClinVar with various classification: one as uncertain in a patient with developmental delay and/or other significant developmental or morphological phenotype (Variation ID: 58862) and another as benign in a patient without evidence of RS (Variation ID: 150074).

In 2007 Simpson et al. detected pseudodicentric chromosome 7 in a patient with features of RS [45,XY, psu dic(7;7)(p22;p22)]. *FAM20C* deletion on both chromosomes 7 was confirmed by FISH (fluorescence in situ hybridization) [4].

487 kbp homozygous deletion in *FAM20C* at genomic position of hg19: 36,480–523,731 was reported by Ababneh et al. in a child with clinical features of RS. Parents were consanguineous and both carried the deletion [1].

Whyte et al. identified heterozygous deletion in exon 3 of *FAM20C* in a mother of two girls with RS phenotype. DNA sequencing in one of the daughters revealed a missense variant in exon 6 of *FAM20C*: c.1094G > A p.Gly365Asp. No other genetic testing has been performed due to DNA degradation. The second girl had not have any genetic testing done. Both girls died and there was no possibility to repeat any tests. However, the authors strongly suspected that both affected girls were compound heterozygotes for *FAM20C* variants [3].

Survival time and severity of presentation in both affected fetuses presented in this report suggest a lethal form of RS (LRS). First affected pregnancy (II) was terminated at 21 weeks of gestation. Pregnancy III resulted in a live-birth, however, the child died after 4 h.

Palma-Lara et al. estimated frequencies of phenotypic features based on the review of 40 cases with LRS. This type of RS is characterised by generalised osteosclerosis, distinct facial features and intercranial calcifications. Among described craniofacial features exophthalmos was present in all patients (100%) followed by midface hypoplasia (95%), depressed nasal bridge (85%), intracerebral calcifications (70%), wide fontanelle, and micrognathia (75%), choanal atresia or stenosis (62%). Palate defects were described in 64% of cases, including cleft (31%) and a high narrow palate. Other significant features included microcephaly, fish-like or tented mouth, and low-set ears. Dental anomalies have not been frequently reported due to early lethality. Skeletal anomalies most frequently regarded long bones and skull sclerosis (90%). Additional frequent features noted by the authors included short neck and thorax. Less common features included shorter limbs, vertebral defects and fractures. Additionally, in 25% of patients structural brain defects have been noted, including cortical dysplasia, cerebellar hypoplasia, gliosis, dysgenesis of corpus callosum and hypophysis. The rarest features included cardiac anomalies, pulmonary hypertension, genito-urinary anomalies and calcification of ovaries, kidneys, liver and adrenal glands [2].

Phenotype in both affected fetuses was consistent with the clinical picture for RS. There were, however, additional features present, not reported previously. They were: single-chamber nasal cavity, narrow septum pellucidum, hyperextensibility of the thumb (‘hitchhiker’s thumb’) in fetus from pregnancy II, and (prenatally) hemorrhages, soft skull bones with lemon sign, shallow eye sockets, followed by (postnatally) large midline bone loss (in forhead – occipital axis), conjunctival hemorrhages and significant dilatation of the lateral brain ventricles in fetus from pregnancy III.

Prenatal diagnosis of RS is challenging and for this reason many cases are only diagnosed postnatally based on characteristic set of clinical features. To the best of our knowledge, several dozen of retrospective cases of RS have been reported and only a few for which diagnosis of RS was made prospectively. We summarized these last cases in Table 1.

**Table 1:** Retrospective and prospective prenatal genetic diagnosis for Raine syndrome – review of clinical and genetic data in previously published cases

Case	References	Prenatal features	Genetic background/cause*. Gene *FAM20C* variants	Clinical diagnosis	Outcome/time of death
1.	2003 Al-Gazali [13]	USS: Short limbs and polyhydramnios.	not tested	postnatal	death; 38 days
2.	2003 Hülskamp [14]	USS (32 weeks): small bi-parietal diameter (BPD), symmetric paraventricular foci of increased cerebral density, carp-shaped mouth, short philtrum, receding mandible. Small thorax compared to the abdomen, fixed and abnormally positioned limbs.	not tested	postnatal	death; 76 min
3.	2003 Hülskamp [14]	USS (23 + 3 weeks): clinical features similar to the case 2 from the same family (above).	not tested	post mortem	termination of preganancy (TOP); 24 weeks
4.	2003 Hülskamp [14]	USS: clinical features similar to the previous two cases from the same family; microcephaly, intracranial calcifications, thoracic hypoplasia and suspected fractures of the long tubular bones.	not tested	postnatal	death; 5 days
5.	2007 Czitayat [15]	USS (12 weeks); nuchal translucency (NT) 1.5 mm USS (19 weeks); no abnormalities detected but left kidney pyelectasis. Retrospectively: flat profile and hypertelorism. USS (35 weeks): abnormal brain with large ill-defined choroid plexus, not well-defined lateral ventricles and echogenic and ill-defined cerebellum. MRI: unusual appearance of the skull vault with reduced transverse diameter in the frontal brain region relative to the interparietal region with narrow transitional zone in the region of the parietal bone. Flat facial profile, very small and hypoplastic nose, hypertelorism, prominent eyes.	not tested	postnatal	stillbirth
6.	2011 Fradin [16]	USS (21 weeks): diffuse hyperechogenicity of the cerebral parenchyma and abnormal flat facial profile. MRI (30 weeks): increased diffused perivascular signals.	c.940C >T, p.P314S	postnatal	non-lethal type in childhood
7.	2011 Fradin [16]	USS: similar features to previously occurred in the family (case 6 above). Polyhydramnios. MRI: cerebral calcifications.	c.940C >T, p.P314S	postnatal	non-lethal type in childhood
8.	2011 Gaigi [17]	USS (34 weeks): periventricular brain hyperechogenicity, facial dysmorphism, lung hypoplasia.	not tested	postnatal	death; 4 h
9.	2011 Kim [18]	USS (22 weeks): bilateral choroid plexus cysts, strawberry-shaped head, echogenic intracardiac focus in the left ventricle. USS (24 weeks): thoracic hypoplasia. USS (33 weeks): growth restriction, intermittent bradycardia, ventricular septal defect, polyhydramnios. 3D USS (33 weeks): abnormal face with bulging eyes, absent nose and triangular shaped mouth.	not tested	postnatal	stillbirth
10.	2014 Mahmood [19]	USS: craniofacial dysmorphism.	c.1135G>A, p.G379R	postnatal	non-lethal type in childhood
11.	2015 Seidahmed [20]	USS (26 weeks): polycystic kidneys, absent nasal bone, clenched hands, polyhydramnios.	NM_020223:c.1225C>T, p.R409C	postnatal	death; 1 day
12.	2018 Tamai [21]	USS (35 weeks): cerebral hyperechogenicity, hypoplastic nose. MRI (36 weeks): flat facial profile and normal brain.	NM_020223.3:c.1219T>G, p.Try407Gly	postnatal	non-lethal type in childhood
13.	2018 Sheth [22]	USS: intrauterine growth restriction (IUGR)	NM_020223.3:c.1228T>A, NP_064608:p.Ser410Thr	postantal	non-lethal type in childhood
14.	2019 Hung [23]	USS (33 weeks): intrauterine fractures.	NM_020223.3:c.1007T>G, p.Met336Arg	postnatal	death; shortly after birth
15.	2020 Eltan [24]	USS: microcephaly, nasal bone agenesis	c.1645 C>T, p.Arg549Trp and c.863+5G>C	postnatal	death; 17 min
16.	2020 El-Dessouky [5]	USS (22 weeks): small head, small cerebellum, intracranial calcifications distributed along the walls of the lateral ventricles, thalamus, and the basal ganglia. Polyhydramnios. 3D USS: mid-face hypoplasia, prominent forehead with bi-temporal narrowing, prominent and wide-spaced eyes, down slanting palpebral fissures, absent nasal bone, depressed nasal bridge, small mouth with down-turned corners, high arched palate, severe micrognathia, low-set ears. Narrow thoracic circumference with rib fractures. Thickening of all the fetal long bones with bilateral mild shortening of the humerus and fracture of the left femur. Family history of polyhydramnios and dysmorphic features on USS in first pregnancy; the child died 65 min after birth.	c.456delC, p.Gly153Alafs*34	prenatal	TOP; 26 weeks
17.	2020 El-Dessouky [5]	USS (24 weeks): small BPD, small head circumference (HC), small cerebellum; intracranial calcifications within ventricular wall, white matter, basal ganglia, thalamus, and cerebellum. Mid-face hypoplasia, prominent forehead with bi-temporal narrowing, prominent and wide-spaced eyes, down-slanting palpebral fissures, absent nasal bone, depressed nasal bridge, small mouth with down-turned corners, high arched palate, severe micrognathia, and low-set ears. Narrow chest. Increased thickening of long bones and mild shortening of the humerus. Scattered calcifications in the kidneys and liver. Dichorionic diamniotic twin pregnancy: second fetus – healthy.	c.905delT, p.Phe302Serfs*35	prenatal	death; 35 min
18.	2020 El-Dessouky [5]	USS (26 weeks): small BPD and HC. Focal calcifications in cerebellum, basal ganglia, thalamus, periventricular white matter and around the ventricular walls. USS 3D: abnormal profile with mid-face hypoplasia, prominent forehead with bi-temporal narrowing, prominent and wide-spaced eyes, down-slanting palpebral fissures, absent nasal bone, depressed nasal bridge, small mouth with down-turned corners, high arched palate, severe micrognathia, and low-set ears. Short and narrow chest. Marked thickening of all long bones with bilateral fractures of relatively short humerus. Calcifications in the liver. Polyhydramnios.	c.1557C>G, p.Tyr519*	prenatal	death; 4 h
19.	2020 Hernández-Zavala [25]	USS (21 weeks): ocular proptosis, hypertelorism and oral anomalies.	No genetic testing in the fetus. NM_020223.3:c.456dupC p.Gly153ArgfsTer56 in the mother NM_020223.3:c.474delC p.Ser159ProfsTer28 in the father	postnatal	TOP; 21 weeks
20.	2020 Mameli [26]	USS: bilateral periventricular hyperechogenicity associated with abnormal cranial conformation.	c.1351G>A, p.Asp451Asn	postnatal	death; shortly after birth
21.	2021 Bajaj [27]	USS (32 weeks): clover-leaf skull, non‐ossified nasal bone, midface hypoplasia, bulging eyelids, irregular contour of long bones.	c.1680C>A, p.Cys560Ter	postnatal	death; 17 min
22.	2021 Lulla [28]	USS (35 weeks): microcephaly with a clover-leaf skull, intracranial calcifications. Marginally small thorax in relation to the abdomen. Osteosclerosis and bone fractures. USS 3D: midface hypoplasia, small anteverted nose, proptosis, everted lips, a small triangular mouth, long philtrum, high arched palate, and hypertelorism. Turricephaly with a narrow forehead. Premature fusion of the coronal sutures.	NM_020223.3:c.1680 C > A p.Cys560*	postnatal	no confirmed data; assumed death
23.	2022 Rameh [12]	USS (24 + 6 weeks): IUGR, microcephaly, calcifications in the choroid plexus and periventricular white matter, exophthalmos, hypertelorism, depressed nasal bridge, midface and thoracic hypoplasia. CT-scan: microcephaly, intracranial calcifications, midface hypoplasia. Previous pregnancy: third-trimester USS: skull bone abnormalities and exophthalmos - an intra-uterine fetal demise.	NM_020223:c.1363+1G>A	prenatal	death; directly after birth
24.	2023 Andrews [6]	USS (26 weeks): calcification in the periventricular white matter and tentorium, micrognathia, exophthalmos, narrow thorax with short ribs.	not tested	postnatal	TOP
25.	2023 Andrews [6]	USS (29 weeks): calcification in the periventricular white matter and tentorium, craniosynostosis, micrognathia, exophthalmos, narrow thorax with short ribs, short femur with irregular contour of long bones and ribs.	not tested	postnatal	TOP
26.	2023 Andrews [6]	USS (20 weeks): calcification in the choroid plexus and periventricular white matter, scalloping of frontal bone, bilateral exophthalmos, hypertelorism, narrow thorax with short ribs. USS 3D: exophthalmos, midface hypoplasia, depressed nasal bridge, micrognathia. Previous pregnancy with multiple calcification in the choroid plexus and exophthalmos on scan - TOP.	p.Pro496SerfsTer80	postnatal	TOP
27.	2024 Verscaj [11]	USS (32 + 4 weeks) and MRI (33 + 5 weeks): multisuture craniosynostosis with protrusion of the brain parenchyma through fontanelles and cranial lacunae, midface hypoplasia, midline cleft lip and palate, absent visualization of the nasal passages with small/abnormal nose; glossoptosis with narrowing of the airway, exorbitism, hypertelorism, small thoracic to abdominal circumference ratio. Polyhydramnios.	NM_020223.4:c.1445G > A p.Gly482Glu	postnatal	death; 1 h

BPD – bi-parietal diameter, CT – computed tomography, HC – head circumference, IUGR – intrauterine growth restriction, MRI – magnetic resonance imaging, NT – nuchal translucency, TOP – termination of pregnancy, USS – ultrasound scan, * – variant nomenclature was taken directly from the referenced publication

El-Dessouky et al. mentioned absence of syndactyly, which helps differentiate RS from other craniosynostoses, including Apert (OMIM 101200), Pfeiffer (OMIM 101600) and Crouzon (OMIM 123500) syndromes [5]. 3D USS, alongside MRI (magnetic resonance imaging) or CT (computed tomography) scans may become a helpful imaging tool supporting invasive prenatal diagnosis methods [5, 12].

Although both fetuses presented in this report shared features that had previously been reported prenatally in association with RS, clinical suspicion of this syndrome has not been made, partially due to its rare occurence. Clinical suspicion of Crouzon syndrome was made during the course of pregnancy III, however, the patient and her partner did not consent for any prenatal testing other than cytogenetic analysis of amniotic fluid samples. Subsequently, an informed consent for retrospective testing of samples from pregnancy II and III was obtained from the patient 12 months after pregnancy III. The prenatal samples were tested as part of a research project. Application of aCGH, NGS and Sanger sequencing led to definitive diagnosis and enabled appropriate genetic counseling for the family.

## Conclusions

It is important to report all cases of Raine Syndrome but especially those with prenatal scan anomalies. It would lead to more accurate identification of this syndrome (as early as at 20 weeks of gestation) and appropriate prenatal diagnosis. Prenatal confirmation of diagnosis would lead to overall better patient care – from comprehensive information provided to pregnant patients, through better management of pregnancy and delivery/termination of pregnancy, to postnatal support and/or palliative care.

## Supplementary Information

Below is the link to the electronic supplementary material.

Supplementary Material 1

## Data Availability

SNV variant was submitted to the LOVD database (SNV ID: 0000952394). https://databases.lovd.nl/shared/variants/0000952394#00007401. Both variants were submitted to the ClinVar database (CNV ID: 4538528 and SNV ID: 4538529). https://www.ncbi.nlm.nih.gov/clinvar/variation/4538528/?term=4538528%5BVariation+ID%5D. https://www.ncbi.nlm.nih.gov/clinvar/variation/4538529/?term=4538529%5BVariation+ID%5D. The datasets used and/or analysed during the current study are available from the corresponding author on reasonable request.

## Abbreviations

aCGH: Array comparative genomic hybridization
ACMG: American College of Medical Genetics and Genomics
BPD: Bi-parietal diameter
CNVs: Copy number variants
CT: Computed tomography
FISH: Fluorescence in situ hybridization
GTG: G-banding using trypsin and Giemsa
IGV: Integrative Genomics Viewer
HC: Head circumference
ISCN: International System for Human Cytogenomic Nomenclature
IUGR: Intrauterine growth restriction
LRS: Lethal form of Rayne syndrome
MRI: Magnetic resonance imaging
N: Normal karyotype
NGS: Next generation sequencing
NT: Nuchal translucency
RS: Rayne syndrome
SNVs: Single nucleotide variants
S.seq: Sanger sequencing
TOP: Termination of pregnancy
USS: Ultrasound scan
